# On the molecular relationship between Hounsfield Unit (HU), mass density, and electron density in computed tomography (CT)

**DOI:** 10.1371/journal.pone.0244861

**Published:** 2020-12-31

**Authors:** Atchar Sudhyadhom

**Affiliations:** 1 Brigham & Women’s Hospital, Boston, MA, United States of America; 2 Dana Farber Cancer Institute, Boston, MA, United States of America; 3 Harvard Medical School, Boston, MA, United States of America; 4 University of California, San Francisco, San Francisco, CA, United States of America; St. Vincent Medical Center, UNITED STATES

## Abstract

Accurate determination of physical/mass and electron densities are critical to accurate spatial and dosimetric delivery of radiotherapy for photon and charged particles. In this manuscript, the biology, chemistry, and physics that underly the relationship between computed tomography (CT) Hounsfield Unit (HU), mass density, and electron density was explored. In standard radiation physics practice, quantities such as mass and electron density are typically calculated based off a single kilovoltage CT (kVCT) scan assuming a one-to-one relationship between HU and density. It is shown that, in absence of mass density assumptions on tissues, the relationship between HU and density is not one-to-one with uncertainties as large as 7%. To mitigate this uncertainty, a novel multi-dimensional theoretical approach is defined between molecular (water, lipid, protein, and mineral) composition, HU, mass density, and electron density. Empirical parameters defining this relationship are x-ray beam energy/spectrum dependent and, in this study, two methods are proposed to solve for them including through a tissue mimicking phantom calibration process. As a proof of concept, this methodology was implemented in a separate in-house created tissue mimicking phantom and it is shown that sub 1% accuracy is possible for both mass and electron density. As molecular composition is not always known, the sensitivity of this model to uncertainties in molecular composition was investigated and it was found that, for soft tissue, sub 1% accuracy is achievable assuming nominal organ/tissue compositions. For boney tissues, the uncertainty in mineral content may lead to larger errors in mass and electron density compared with soft tissue. In this manuscript, a novel methodology to directly determine mass and electron density based off CT HU and knowledge of molecular compositions is presented. If used in conjunction with a methodology to determine molecular compositions, mass and electron density can be accurately calculated from CT HU.

## Introduction

Determination of physical/mass density and electron density are the cornerstone of photon and charged particle dose calculation for heterogeneous (non-water) tissues. In the case of photon dose calculation, electron density values are used to scale effective pathlength to account for tissue inhomogeneities in the commonly used superposition/convolution algorithm [1]. For the case of particle therapy, the range of a particle can be determined by the Bethe-Bloch equation, which is linear with electron density. Thus, errors and uncertainties in electron density are directly related to range uncertainty, a fundamental problem limiting the clinical efficacy of proton therapy. In addition, for Monte Carlo based dose calculation algorithms, mass density information explicitly separate from the elemental composition may be required for accurate dose calculation.

The current clinical standard to determine mass density, *ρ*, and electron density relative to water, *ρ*_*e*_, is through conversion of single energy kilovoltage computed tomography (kVCT) scan data. In clinical practice, a relationship between Hounsfield Units (HU)/CT numbers and the requisite type of density (either mass and/or electron) is determined through a calibration process involving a tissue surrogate phantom. Subsequent scans rely on this predetermined relationship to correlate voxel-by-voxel HU values in patient scans to mass density and electron density, assuming consistent HU values over time. For kVCT, HU is non-linear and non-bijective with either type of density as average atomic number and elemental composition varies by tissue [2]. Due to the non-bijective nature of this relationship, the exact composition of the tissue surrogate phantom will bias the accuracy of subsequent HU to electron density conversions in human tissues with lower energy CT (kVCT) being more sensitive to exact elemental composition than higher energy CT [3]. As an example of this, uncertainties in mass density [4] have been found to produce significant changes (of up to 12.5%) to the Monte Carlo calculated dose distribution for physically reasonable perturbations of the CT to density conversion tables.

As opposed to kVCT, megavoltage CT (MVCT) is considered to be more linear with electron density due to Compton scattering being the primary mode of photon interaction and thus less dependent on atomic composition [5, 6]. In prior studies [7–9], high-energy x-rays beams, in which Compton scattering dominates, has been found to produce images that can be converted to high accuracy electron density. Even for centers with MVCT capability, kVCT is still the preferred imaging methodology due to its lower noise, greater soft tissue, and strong bone contrast not found in MVCT scans [10, 11]. With the requirements for MVCT imaging being, in general, restrictive due to costly equipment as an imaging device, some groups have proposed an intermediate energy, orthovoltage CT (OCT) modality [3] that might be more feasible for clinical imaging. In the work of Yang *et al*. [3], they found that OCT was less sensitive to beam hardening artifacts and produced a more linear and accurate HU to electron density relationship than kVCT and more in line with trends found for MVCT. Yet higher energy CT modalities, such as both OCT and MVCT, are not widely available CT products in the vast majority of clinics. Thus, understanding and mitigating the source of inaccuracies and degeneracy in the relationship between kVCT HU and electron density is crucial to dose calculation accuracy for most centers and is particularly important for proton therapy centers.

In this manuscript, the fundamental basis for the relationship between HU, mass density, and electron density was explored considering not only the underlying physics but also related chemistry and biology. In doing so, it was discovered that kVCT HU exhibits a highly non-bijective relationship with electron density, when normalized for mass density. Though non-bijective, this relationship exhibits a multi-dimensional nature that is a function of molecular and tissue composition which, if known, can be used to improve the accuracy of mass and electron density determination. As a proof of principle, it is shown in a tissue mimicking phantom that mass density and electron density can be uniquely determined by CT HU and knowledge of the respective tissue/voxel’s molecular composition.

## Materials and methods

### Theoretical basis for the relationship between HU, mass density, and electron density

In order to show the mathematical relationship between these various quantities, relevant quantities of interest in this work are defined. First, HU is defined as:

$$HU=1000\times \left(\frac{\mu_{x}-\mu_{water}}{\mu_{water}}\right)$$ (1)

where *x* is either some heterogenous collection of atoms and molecules as in a type of tissue or even spatially as within a voxel. The total linear attenuation coefficient of the tissue and/or region, *x*, at a given x-ray energy (or energy spectrum) is defined as:

$$\mu_{x}=\rho_{x}\sum_{i}\left(\omega_{i}\frac{\mu_{i}}{\rho_{i}}\right)$$ (2)

where *ρ*_*x*_ is the mass density of *x*, *i* are all the elemental constituents of *x*, and *ω*_*i*_ are the percentages by mass for each element of *x*. It is also helpful to define a mass normalized version of Eq 2, the mass attenuation coefficient, *μ*_*x*_/*ρ*_*x*_. As the mass density of a material/tissue is not fundamental property of that material, it is helpful to define a new quantity, a mass density normalized variant of HU, HU_⍴_, as:

$${HU}_{\rho }=1000\times \left(\frac{\mu_{x}/\rho_{x}-\mu_{water}/\rho_{water}}{\mu_{water}/\rho_{water}}\right)$$ (3)

such that the attenuation coefficient found in HU was replaced with mass attenuation coefficient and *x* is a particular biological molecule or tissue type of interest. From Eqs 1 and 3, HU_⍴_ can be solved as a function of HU and *ρ* such that:

$${HU}_{\rho }=[\left(HU+1000\right)/\rho ]-1000$$ (4)

and similarly, *ρ*_*x*_ can be written as a function of HU_⍴_ and HU such that

$$\rho =\left(HU+1000\right)/\left({HU}_{\rho }+1000\right).$$ (5)

In the later parts of this work, Eq 5 is used to calculate mass density from HU. It is noted that HU_⍴_ is fundamental property of a material/tissue’s attenuation of a given x-ray beam as it is only a function of elemental/molecular constituents and x-ray beam energy. Thus, Eq 5 provides a relationship of HU to mass density that can, in principle, be calculated *a priori* from fundamental physics and compositional knowledge of a material/tissue. Finally, electron density is defined as:

$$\rho_{e}=\rho \left[\sum_{i}\left(\omega_{i}\frac{Z_{i}}{A_{i}}\right)\right]{\left(\frac{Z}{A}\right)}_{water}^{-1}.$$ (6)

### Understanding the molecular and tissue specific relationship between HU, mass density, and electron density

Biological tissues are generally composed of water, lipids, proteins, and minerals/hydroxyapatite with the exact proportions dependent on the particular tissue/organ [12, 13]. Four-component models of molecules in the human body have been used previously in the study of human tissues [12, 13] and are assumed, in this work, as the model for naturally occurring biological tissues. Thus, human tissues were considered as some linear combination of all these molecules. Using the NIST XCOM database [14], the mass attenuation coefficient (*μ*/*ρ*) was calculated for all major molecules that fit within the aforementioned four categories (water, lipids, proteins, and minerals) using an estimated x-ray energy spectrum for a 120 kVp kVCT and a 3.5MV MVCT x-ray beams.

**Fig 1:**
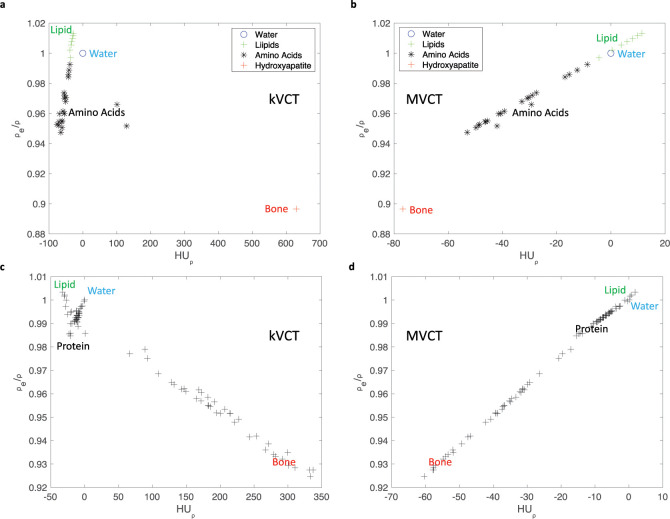
Calculated mass density normalized HU, HU⍴, were plotted against mass density normalized electron density, *ρ*_*e*_/*ρ*. For biologically relevant molecules (A) kVCT and (B) MVCT energies. For human tissues at (C) kVCT and (D) MVCT. Molecule types are indicated by symbols, colors, and with labels in panels A and B. For panels C and D, tissue types contained a wide range of molecules although trends towards higher lipid, water, and protein content are labelled with text in the figure.

To determine HU_⍴_, the energy spectrum of these two imaging beam energies was modeled to calculate total linear attenuation and mass attenuation coefficients for all elements. There exist multiple developed methodologies [15, 16] to calculate and/or determine x-ray spectra for diagnostic kilovoltage beam energies as in kVCT. For the modeling of a kVCT 120kVp beam, the TASMICS methodology of Punnoose *et al*. [15] was employed with a 120 kVp Boone/Fewell tube with an assumed equivalent of 1.25mm of Cu filtration. As, to the best of knowledge of the author, there does not exist an analogous MVCT beam model methodology (as of SPEKTR), the MVCT energy distribution was modeled with a gamma probability density function (PDF) with parameters fit to the MVCT energy spectrum found in Jeraj *et al*. [17] producing a weighted average energy of 0.86 MeV. HU_⍴_ and mass density normalized electron density relative to water, *ρ*_*e*_/*ρ*, were calculated for all the molecules modeled and the results were plotted in Fig 1. As can be seen in Fig 1A, for kVCT the majority of organic molecules (lipids and proteins) lay along a line with water neighboring this regime. This relationship suggests that a linear combination of any of these organic molecules within tissues will have a relatively linear HU_⍴_ relationship with *ρ*_*e*_/*ρ*. If the case in which water may be a constituent of these tissues is considered, the errors can increase as there is no longer an accurate one-to-one relationship to map HU_⍴_ to *ρ*_*e*_/*ρ*. Which is to say, absent of mass density data, there does not exist a bijective relationship between HU and *ρ*_*e*_. Errors of 1–2% in *ρ*_*e*_/*ρ* may result due to a degeneracy of HU_⍴_ for any material that contains a combination of organic and water molecules. When considering the case of boney tissues which may include some linear combination of all types of molecules, this degeneracy of HU_⍴_ to *ρ*_*e*_/*ρ* increases significantly as hydroxyapatite is distant from the linear relationship found among organic molecules. At HU_⍴_ values around 0 and considering all possible molecules, the relationship with *ρ*_*e*_/*ρ* is much less clear resulting in a range of values that vary by up to 7%. For MV energies as in Fig 1B, this relationship appears to be linear for most organic molecules and water indicating that a linear combination of molecules is more likely to produce a one-to-one relationship between HU_⍴_ and *ρ*_*e*_/*ρ*.

Biological tissues are a complex combination of biological molecules (water, mineral, and organic molecules). As such, it is expected that biological tissues exhibit a distinct but related relationship between HU_⍴_ and *ρ*_*e*_/*ρ* that depends on prevalence of the biological molecules that comprise them. Thus, the HU_⍴_ versus *ρ*_*e*_/*ρ* relationship for biological tissues was explored for soft tissues and boney tissues using elemental compositions reported from tissues in the literature [18–21]. Those results are shown in Fig 1C and 1D. As anticipated, the relationship between HU_⍴_ and *ρ*_*e*_/*ρ* is non-bijective for kVCT. For kVCT (as in Fig 1C), it is noted that tissues tend to distribute along 4 major axes: lipid heavy (as in yellow marrow and adipose), water heavy (as in urine, blood and cerebrospinal fluid), protein heavy (as in muscle, skin, and connective tissue), and mineral/hydroxyapatite heavy (as in various types of bone). While tissues tend to group into these four major axes, it should be noted that tissues, in general, tend to not be predominantly composed of any only one type of molecule. For example, the “standard” cortical bone of ICRU 44 is approximately 60% mineral/hydroxyapatite, 30% protein, and 10% water. Most interestingly, within boney tissues there tends to be two major types: more proteinaceous and more lipid/water heavy and visualized in Fig 1C as two separate set of points starting at HU_⍴_ > 50. For MVCT (as in Fig 1D), it is noted that a relatively one-to-one (linear) relationship exists between HU_⍴_ and *ρ*_*e*_/*ρ*.

In radiotherapy physics clinical practice, the degeneracy that’s exemplified in Fig 1C is not as obvious (and often overlooked) due to many biological tissues tending to have mass densities that are dissimilar from each other. Calibration of HU to *ρ*_*e*_ is then completed by assuming nominal tissue elemental compositions and densities. The integration of nominal density and assumed tissue elemental compositions may appear to reduce this degeneracy although groups have seen that a significant degeneracy still exists for kVCT [2, 3]. While it is anecdotally held that density uniquely varies with tissue type, density is not a fundamental property of any type of material or tissue. Soft tissues are intrinsically elastic with mass density changes possible over time. In addition, there are many tissues that have similar densities such as low-density bone (trabecular) and high-density soft tissue (muscle) with vastly differing composition. Thus, assuming fixed densities for specific types of tissues will inevitably lead to significant uncertainties in the HU to *ρ*_*e*_ conversion process for kVCT.

### Determination of mass density and electron density by HU and known molecular composition

In the prior section, it was shown that, in absence of mass density information, there appears to be a multi-dimensional relationship between HU_⍴_ and *ρ*_*e*_/*ρ* that could be further predicted based on molecular composition. This relationship appears to be linear relative to each of those molecularly based dimensions. To define this relationship between HU, mass density, and electron density, were analyzed in a subset of the well-studied tissues of ICRU 44 [21] and the related works of White *et al*. [19, 22] and Woodard and White [20, 23] that had both elemental and molecular compositions. In this section, the empirical derivation of parameters for the relationship between mass density, electron density, HU, and molecular composition is shown.

First, HU_⍴_ is defined as a function of molecular composition. Reorganizing the sum of elements as in Eq 2 as a sum of those molecular constituents, the mass attenuation coefficient equation can be rewritten as:

$$\frac{\mu_{x}}{\rho_{x}}=\sum_{i}\left(\omega_{i}\frac{\mu_{i}}{\rho_{i}}\right)=\omega_{water}\frac{\mu_{water}}{\rho_{water}}+\omega_{lipids}\frac{\mu_{lipids}}{\rho_{lipids}}+\omega_{proteins}\frac{\mu_{proteins}}{\rho_{proteins}}+\omega_{minerals}\frac{\mu_{minerals}}{\rho_{minerals}}.$$ (7)

where *ω* is the percentage by mass of the respective molecule within *x* and such that ∑_*i*_*ω*_*i*_ = 1. Using the result of Eq 3 in Eq 4, HU_⍴_ can be written as a function of molecular constituents such that:

$$\begin{array}{c} {HU}_{\rho }=\left(\frac{1000}{\mu_{water}/\rho_{water}}\right)\left((\omega_{water}-1)\frac{\mu_{water}}{\rho_{water}}+\omega_{lipids}\frac{\mu_{lipids}}{\rho_{lipids}}+\omega_{proteins}\frac{\mu_{proteins}}{\rho_{proteins}}+\omega_{minerals}\frac{\mu_{minerals}}{\rho_{minerals}}\right)\\ =\omega_{lipids}\alpha_{1}+\omega_{proteins}\alpha_{2}+\omega_{minerals}\alpha_{3} \end{array}$$ (8)

with $\alpha_{i}=1000\times \left(\frac{\mu_{i}}{\rho_{i}}\times \frac{\rho_{water}}{\mu_{water}}-1\right)$ for *i* = [1,2,3] for lipids, proteins, and minerals, respectively. It is noted that HU_⍴_ is a multiple linear equation that is a function of each *ω*, the percent by mass of each molecular component, and constants that are a function of mass attenuation and x-ray beam energy spectrum. Second, it can similarly be shown that *ρ*_*e*_/*ρ* can also be expressed as a linear sum of molecular composition and constants such that:

$$\begin{array}{c} \rho_{e}/\rho =\left[\omega_{water}\frac{Z_{water}}{A_{water}}+\omega_{lipids}\frac{Z_{lipids}}{A_{lipids}}+\omega_{proteins}\frac{Z_{proteins}}{A_{proteins}}+\omega_{minerals}\frac{Z_{minerals}}{A_{minerals}}\right]{\left(\frac{Z}{A}\right)}_{water}^{-1}\\ =1+\omega_{lipids}\beta_{1}+\omega_{proteins}\beta_{2}+\omega_{minerals}\beta_{3} \end{array}$$ (9)

with $\beta_{i}=\left(\frac{Z_{i}}{A_{i}}\times \frac{A_{water}}{Z_{water}}-1\right)$ for *i* = [1,2,3] for lipids, proteins, and minerals, respectively. Finally, electron density, *ρ*_*e*_, can be defined as an explicit function of HU and molecular compositions by combining Eqs 8 and 9 with Eq 5 to get:

$$\rho_{e}=(HU+1000)\times \frac{1+\omega_{lipids}\beta_{1}+\omega_{proteins}\beta_{2}+\omega_{minerals}\beta_{3}}{\omega_{lipids}\alpha_{1}+\omega_{proteins}\alpha_{2}+\omega_{minerals}\alpha_{3}+1000}$$ (10)

To determine *β*_*i*_, *ρ*_*e*_/*ρ* was calculated in the tissues of ICRU 44 [21] and the related works of White *et al*. [19, 22] and Woodard and White [20, 23] from their tabulated elemental compositions and regressed those values against their respective molecular (lipid, protein, and mineral) compositions. From those data, the respective parameters for *β*_*i*_ were determined to be: 0.00499, -0.0419, and -0.113 for lipids, proteins, and minerals, respectively, with an R^2^ = 0.998 and an RMS error of < 0.001. As *ρ*_*e*_/*ρ* is linear with these parameters, the function’s sensitivity to uncertainties in molecular composition knowledge can be directed calculated and note errors of 0.000050, 0.00042, 0.0011, per percent change in lipids, proteins, and minerals, respectively. In addition, *β*_3_ was calculated by assuming hydroxyapatite as the sole mineral and determined the *β*_3_ value to be -0.103, and in line with the value of -0.113 from the empirical fit.

**Fig 2:**
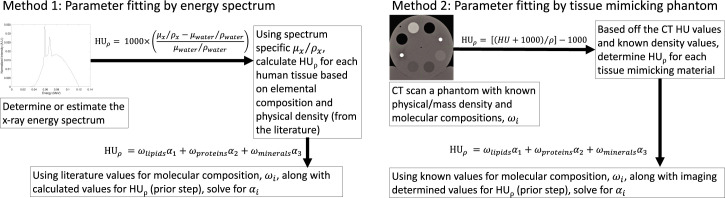
Workflow for the two proposed methods to determine values for *α*_*i*_ parameters needed to calculate HU⍴. In method 1 (left), an energy spectrum must be explicitly determined *a priori* whereas in method 2 (right) these parameters are obtained by imaging a phantom.

The *α*_*i*_ parameters needed to calculate HU_⍴_ (as in Eq 8) are energy dependent and must be determined for a given energy spectrum. As such, they can be calculated through direct knowledge of the energy spectrum of the x-ray imaging beam of interest or indirectly measured. In this work, the *α*_*i*_ parameters of Eq 8 were determined by two methods: 1) using the x-ray energy spectrum to calculate HU_⍴_ in Eq 3 and 2) using Eq 4 in a phantom with known mass densities to calculate HU_⍴_ then in either case, *α*_*i*_ parameters would be determined by fit of known molecular compositions (*ω*_*i*_) for water, lipid, protein, and minerals. By this first method, two energy spectrums: 1) for a 120 kVp kVCT imaging beam and 2) a 3.5MV MVCT imaging beam with energy spectra created as discussed in Section 2.2 were, separately, investigated. Then HU_⍴_ was calculated in all tissues of ICRU 44 [21] and the related works of White *et al*. [19, 22] and Woodard and White [20, 23] that had both elemental and molecular compositions tabulated. With these values of HU_⍴_ calculated by elemental composition, the *α*_*i*_ parameters were empirically determined by a multi-linear regression with their respective known molecular compositions, *ω*_*i*_, for each tissue type as given by values in the literature. For kVCT, the *α*_*i*_ parameter values of -35.9, -54.5, and 605.3 were obtained for lipid, protein, and minerals, respectively. For MVCT, the *α*_*i*_ parameter values of 3.3, -42.4, and -86.5 were obtained for lipid, protein, and minerals, respectively. In the second method, *α*_*i*_ parameters were determined from a CT scan of a multiple tissue mimicking materials phantom with known mass density. As it is typically difficult to know/determine the x-ray spectrum, this second method may be preferred in many cases. For this method, HU_⍴_ can be calculated by Eq 4 and fit to determine the *α*_*i*_ parameters through a multi-linear regression with known molecular compositions, *ω*_*i*_, for each synthetic tissue type. The workflow of these two methods developed in this work to determine mass density and electron density from HU and knowledge of molecular compositions is shown in Fig 2. This methodology and proof of principle results are further discussed in the next section.

### Explicit calibration of phantom HU data to mass density and electron density

**Fig 3:**
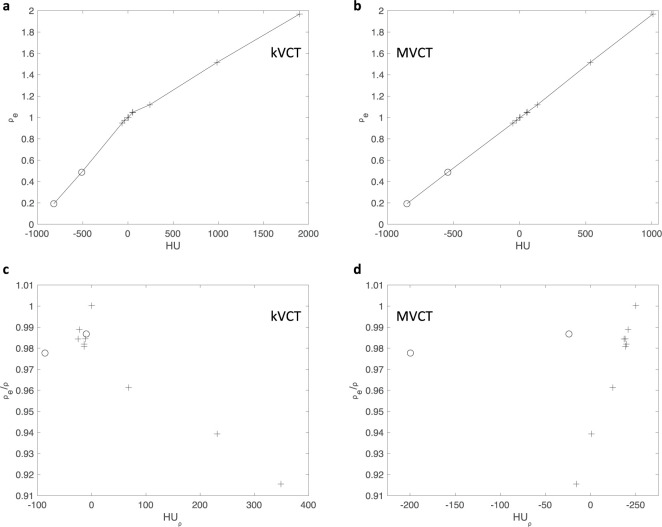
HU plotted against *ρ*_*e*_. For (A) kVCT, (B) MVCT, and HU_⍴_ plotted against *ρ*_*e*_/*ρ* for (C) kVCT, (D) MVCT for a CIRS tissue surrogate phantom with various tissue mimicking materials and liquid water. Circles (o) represent lung mimicking materials while plus signs (+) represent other tissue mimicking materials (including synthetic soft tissue, liquid water, and synthetic bone).

In this study, a Siemens Somatom CT scanner (Somatom; Siemens, Munich) was used to create kVCT images of phantoms. Scans were acquired at 120 kVp using a helical acquisition at an approximate resolution of 1 x 1 x 1 mm in the axial plane (512 x 512 matrix). MVCT imaging (3.5 MV) of phantoms and patients were completed on an Accuray TomoHD system using the “fine” pitch mode with reconstructed slices of 1 x 1 x 1 mm in the axial plane (512 x 512 matrix). Conventional HU to *ρ*_*e*_ calibration curves from a CIRS tissue surrogate phantom are shown in Fig 3. It should be noted that tissue surrogate phantoms (such as the CIRS) are not composed of the biological molecules (water, lipid, proteins, and minerals) typically found in actual tissues. Instead, these phantoms tend to mimic tissues by having similar effective atomic numbers and nominal mass density. Despite that, similar trends in non-linearity for kVCT (Fig 3A) between synthetic soft tissue, liquid water, and synthetic bone materials exist as in the analysis seen in Fig 1. The MVCT (Fig 3B) calibration curve is fairly linear with minimal differences in linearity for most synthetic tissue types and liquid water.

HU_⍴_ and *ρ*_*e*_/*ρ* were also calculated for this tissue mimicking phantom and plotted in Fig 3C and 3D for kVCT and MVCT, respectively. From these plots, it can be seen that the lung mimicking materials do not well align with the expected trend for the tissue like materials as in Fig 1C and 1D. For the MVCT data as in Fig 3D, it can be seen that the majority of materials have a linear trend that the lung mimicking materials do not follow. Upon examination of the elemental material composition of these two tissue mimicking materials, it was found that they were the only materials with significant amounts of chlorine (≥ 1%) which is not typical of biological tissues and may, in part, account for their deviations from biologically expected properties. Thus, these two lung tissues were excluded from the additional analyses as their significant deviations away from biological compositions are not well modeled in this work.

With HU_⍴_ and molecular compositions known for a set of phantom materials, the *α*_*i*_ parameters of Eq 8 can be determined (as in method 2 of Fig 2, right). As the compositions of the CIRS tissue surrogate phantom are not biological molecules, it is not possible to actually know the molecular composition of the synthetic tissues in terms of biological molecules (water, lipids, proteins, and minerals). As a proof of principle, it was assumed the composition of the phantom materials to be similar to that of nominal tissue compositions as in ICRU 44 [21] and the related works of White *et al*. [19, 22] and Woodard and White [20, 23] with the specific assumed compositions shown in Table 1. For kVCT, the *α*_*i*_ parameter values of -50.8, -58.1, and 604.8 were obtained for lipid, protein, and minerals, respectively. For MVCT, the *α*_*i*_ parameter values of 4.5, -54.7, and -77.5 were obtained for lipid, protein, and minerals, respectively. These values are in line with values obtained by method 1 (assuming x-ray energy spectrums for kVCT and MVCT). As an additional check of this methodology, *α*_3_ (for minerals) was calculated using the assumed energy spectrum for both kVCT and MVCT assuming hydroxyapatite as the sole molecule in minerals and obtained the values of 629.9 and -76.7, respectively, which is, again, in line with both method 1 and 2 values for *α*_3_ for kVCT and MVCT.

**Table 1:** Tabulated assumed molecular compositions, manufacturer provided *ρ*, and calculated *ρ*_*e*_ for the calibration phantom.

	Water (%)	Lipid (%)	Protein (%)	Minerals (%)	*ρ* (g/cc)	*ρ*_*e*_	HU(kV)	HU_ρ_(kV)	HU(MV)	HU_ρ_(MV)
Adipose	30	55	15	0	0.958	0.947	-63	-22	-50	-9
Breast	50	25	25	0	0.989	0.974	-31	-20	-24	-13
Liquid Water	100	0	0	0	1.000	1.000	0	0	0	0
Solid Water	100	0	0	0	1.016	1.000	2	-14	4	-12
Muscle	75	5	20	0	1.067	1.047	49	-17	55	-12
Liver	75	5	20	0	1.069	1.050	54	-14	58	-11
Trab Bone	10	40	30	20	1.164	1.119	247	71	134	-26
Dense Bone (800)	5	20	35	40	1.613	1.515	910	184	534	-49
Dense Bone (1750)	3	2	40	55	2.150	1.968	1865	333	1008	-66

HU(kV) and HU(MV) were determined by kVCT and MVCT imaging of this phantom with HU_ρ_(kV) and HU_ρ_(MV) calculated by Eq 4.

### Creation of a tissue mimicking phantom of known composition, *ρ*, and *ρ*_*e*_

As a proof of principle of *ρ* and *ρ*_*e*_ determination, a phantom was developed to specifically mimic the elemental and molecular composition of tissues but not necessarily be of nominal mass density. This tissue mimicking phantom was created in-house by mixing known compositions of distilled water, lipid (coconut oil), protein (porcine gelatin), and minerals (pure molecular hydroxyapatite) using a process previously developed in Scholey *et al*. [24]. The composition of each tissue mimicking material in the phantom is shown in Table 2 and were designed to be similar in composition to ICRU 44 [21] tissues for the respective tissues. Constituents of each mixture were carefully weighed using a high-precision scale (Practum313-1S, Sartorius Biotech, Germany). The skin mimicking material was created by mixing fixed (by mass) amounts of gelatin with hot water until dissolved and allowing for solidification. Muscle and adipose phantoms were created similarly but including coconut oil (adipose solutions were created separately for each container to prevent premature congealing of the predominately oil mixture). Spongiosa phantoms were created in a manner similar to skin phantoms but included hydroxyapatite powder. To determine mass density values of each phantom, volume and mass were determined using volumetric pipettes and the high precision scale. As gelatin and coconut oil are not pure molecular compounds (but rather a mixture of multiple molecules), elemental composition of these substances was determined at a specialized microanalytical facility via carbon, hydrogen, nitrogen, and oxygen (CHNO) combustion analysis. Measured mass density and known elemental compositions were used to determine a ground truth *ρ*_*e*_ for each tissue mimicking material. Error in the determination of mass density and elemental composition were propagated to determine uncertainties in the values of *ρ*_*e*_. Using this phantom, CT images at kVCT and MVCT were acquired and used to determine HU. These HU values were used to calculate *ρ* and *ρ*_*e*_ by CT imaging using two different methodologies: 1) piecewise linear interpolation of HU by CT imaging based on the calibration curves for *ρ* and *ρ*_*e*_ or 2) by Eq 5 and Eq 10 using known water, lipid, protein and mineral compositions, HU, and empirically derived parameters for *α*_*i*_ and *β*_*i*_.

**Table 2:** Tabulated compositions and calculated *ρ*_*e*_ by imaging for synthetic tissue phantoms.

Tissue Type	Water (%)	Lipid (%)	Protein (%)	Minerals (%)	*ρ* (g/cc)	*ρ*_*e*_	*ρ*(*kV*_1_)	*ρ*_*e*_(*kV*_1_)	*ρ*(*kV*_5_)	*ρ*_*e*_(*kV*_5_)	*ρ*(*MV*_1_)	*ρ*_*e*_(*MV*_1_)	*ρ*(*MV*_5_)	*ρ*_*e*_(*MV*_5_)
Skin	75	0	25	0	1.060 ± 0.002	1.048 ± 0.002	1.052	1.033	1.053	1.042	1.065	1.045	1.068	1.057
							(-0.7%)	(-1.4%)	(-0.6%)	(-0.5%)	(0.5%)	(-0.3%)	(0.8%)	(0.8%)
Muscle	75	5	20	0	1.045 ± 0.002	1.036 ± 0.002	1.034	1.017	1.035	1.027	1.051	1.032	1.051	1.042
							(-1.0%)	(-1.9%)	(-0.9%)	(-0.9%)	(0.6%)	(-0.4%)	(0.6%)	(0.6%)
Adipose	47	49	4	0	0.953 ± 0.002	0.955 ± 0.002	0.941	0.931	0.946	0.947	0.970	0.958	0.960	0.961
							(-1.2%)	(-2.5%)	(-0.7%)	(-0.9%)	(1.8%)	(0.3%)	(0.7%)	(0.6%)
Spongiosa	27	47	13	13	1.060 ± 0.002	1.044 ± 0.002	1.099	1.071	1.065	1.046	1.055	1.036	1.059	1.041
							(3.6%)	(2.6%)	(0.4%)	(0.2%)	(-0.4%)	(-0.8%)	(-0.1%)	(-0.3%)

Ground truth *ρ* and *ρ*_*e*_ values were calculated with noted uncertainties and used as a basis of comparison for the two other methods of calculating *ρ* and *ρ*_*e*_ by imaging. For *ρ* and *ρ*_*e*_ by imaging (either kV or MV CT), two methods were used in its determination: 1) piecewise linear (1-dimensional) direct interpolation of HU from CIRS tissue surrogate phantom imaging data (*kV*_1_/*MV*_1_) and 2) calculation by HU and known molecular composition (water, lipid, protein, and minerals) (*kV*_5_/*MV*_5_). Errors compared with ground truth for each imaging determined value of *ρ* and *ρ*_*e*_ are shown in parentheses.

## Results

### Evaluation of *ρ* and *ρ*_*e*_ accuracy in a tissue mimicking phantom

The accuracy of the classical method for determining *ρ* and *ρ*_*e*_ and this newly proposed method was assessed within a phantom that was specifically designed to mimic tissues in elemental and molecular composition but that did not necessarily possess ideal nominal mass densities. The densities within this phantom for skin, muscle, adipose, and spongiosa were 1.060 ± 0.002, 1.045 ± 0.002, 0.953 ± 0.002, and 1.060 ± 0.002 g/cc compared with ICRU 44 [21] nominal densities of 1.09, 1.05, 0.950, and 1.18 g/cc, respectively. The creation of a phantom whose mass density differs from nominal mass density provided a unique opportunity to examine the influence mass density has on the imaging determined accuracy for *ρ* and *ρ*_*e*_ using empirically derived values of method 2 (using a CT scan of the CIRS synthetic tissue phantom). Computed *ρ* and *ρ*_*e*_ values were tabulated in Table 2 with errors for each method compared with *ρ* and *ρ*_*e*_ calculated by known composition/density are noted in parentheses. In the case of kVCT, significant errors in both *ρ* and *ρ*_*e*_ as determined by the standard clinical method, linear piecewise interpolation, were found. MVCT determined *ρ*_*e*_ values were accurate with errors < 1% for either method. For kVCT, the piecewise linear approach proved to be less accurate with errors of around 1–3% for *ρ* and *ρ*_*e*_ among the phantom materials created. In contrast, the multiple linear regression method using kVCT HU values proved to be accurate, with errors of < 1% for all synthetic tissue types. It is likely that the errors measured in this study include errors in determination of the empirically derived parameters, *α*_*i*_. As opposed to the synthetic tissue electron density calibration phantom used in this study, the ideal calibration phantom for this work would be one composed of actual biologically based molecules (water, lipids, proteins, and minerals) with well-known and qualified compositions for each type of molecule. Calibration using such a phantom may help to further minimize these errors.

## Discussion

In this work, the biology, chemistry, and physics that underlay the relationship between HU, *ρ*, and *ρ*_*e*_ in CT imaging were studied. In doing so, the goal was to not only understand this complex relationship but also learn to make it more accurate. To uncover the dependences in this relationship, mass density was removed from HU and *ρ*_*e*_ to examine how these quantities relate if they were independent of mass density. For kVCT, the relationship between HU_⍴_ and *ρ*_*e*_/*ρ* was found to be highly non-linear with a dependence not only on tissue type but specifically on the molecular constituents (water, lipid, protein, and minerals) that comprise them. Based off this information, a newly developed theory and model was created to resolve the dependence that molecular composition has on the relationship between HU, *ρ*, and *ρ*_*e*_. This theoretical model to convert HU to *ρ* and *ρ*_*e*_ was tested in a phantom where elemental/molecular compositions, mass density, and electron density were well quantified. In this phantom, the accuracy of these methods to calculate *ρ* and *ρ*_*e*_ by HU was evaluated. It was found to be possible to accurately (with errors < 1%) calculate *ρ* and *ρ*_*e*_ by MVCT (by both models) and by kVCT with the theoretical basis and model presented in this work. In comparison, kVCT conversion of HU to *ρ* and *ρ*_*e*_ by the clinical standard, piecewise linear interpolation, were generally much less accurate with errors of up to 3.6% and 2.6% for bone *ρ* and *ρ*_*e*_, respectively. While it is possible to know the chemical composition of phantoms to great detail, it is often, unfortunately, not as simple in *in vivo* studies.

While methods to determine molecular composition were not implemented or investigated in this work, there are, potentially, many methods that could be used to either assume or determine the molecular composition of tissues. Recently, spectral CT with photon counting detectors has been devised as a method to discriminate water, fat, and calcium in tissues [25]. Another potential methodology is to assume the molecular composition of tissues based off their nominal/average values for that organ. To employ such a methodology, it may be useful to first complete tissue segmentation using one of the growing number of accurate machine learning based methods [26]. Then, the assumption of nominal molecular compositions may be a reasonable one. Generally, for soft tissues, compositions vary within 5–10% from a given mean value indicating that molecular tissue compositions for a given organ may vary but that the variation may not be that large [19, 20, 22, 23]. Muscle, for example, is reported to have ranges of 68.9%– 80.3%, 2.2%– 9.4%, and 12.9%– 20.0% for water, lipid, and protein, respectively [23]. Similarly, the majority of adipose tissue data lie within 10%– 30%, 70%– 90%, and 0%– 12% for water, lipid, and protein, respectively. Of note, this relationship appears to be age dependent and appears to become more consistent for adults [22]. The sensitivity of this model to voxel specific composition uncertainties was tested with results shown in Table 3. Compositions of 100% pure molecules were assumed and errors were calculated by simulating a 10% error from a separate molecule type. For all soft tissues, tissue composition uncertainties of 10% (equivalent to the natural variation found in soft tissue molecular composition) from the assumed composition resulted in < 0.7% error in both *ρ* and *ρ*_*e*_. For boney tissues, errors due to uncertainties in mineral composition may be more significant with errors of up to 7.6% occurring in situations in which the error in the assumed mineral content was 10%, relative to the tissue’s actual composition. From the works of White *et al*. [19, 22] and Woodard and White [20, 23], it can be seen that adult cortical bone compositions are fairly stable and consistent and thus uncertainties in mineral composition may be much lower than the 10% modeled in this work. Though, in general, more accurate methods to determine mineral content in tissues may be necessary to limit the uncertainty in *ρ* and *ρ*_*e*_ calculation for boney tissues.

**Table 3:** Sensitivity analysis of the HU and molecular composition to *ρ* and *ρ*_*e*_ conversion process for kVCT using *α*_*i*_ values determined by method 2 (CT calibration phantom).

Assumed Pure Composition	Simulated 10% Error	Error in *ρ* (%)	Error in *ρ*_*e*_ (%)
Water	Lipid	-0.5	-0.6
Water	Protein	-0.6	-0.2
Lipid	Water	0.5	0.6
Lipid	Protein	-0.1	0.4
Protein	Water	0.6	0.2
Protein	Lipid	0.1	-0.4
Water	Minerals	5.7	6.8
Lipid	Minerals	6.5	7.6
Protein	Minerals	6.6	7.3
Minerals	Water	-3.9	-5.2
Minerals	Lipid	-4.3	-5.6
Minerals	Protein	-4.3	-5.1

Compositions of 100% pure molecules (as noted in the left most column) were assumed and errors were calculated by simulating a 10% error from a separate molecule type (2^nd^ column from the left).

The relationship between kVCT HU and *ρ* and *ρ*_*e*_ has historically been modeled as linear or piecewise linear. In reality, this relationship is complex without an explicit one-to-one relationship in the conversion of HU to *ρ* or *ρ*_*e*_. As conversion of HU to *ρ* and *ρ*_*e*_ is critically important in radiotherapy dose calculation, several methodologies have been proposed to improve the accuracy of the conversion of HU to *ρ*_*e*_, *ρ*, and stopping power. Dual energy CT (DECT) has recently been proposed to provide more accurate estimates of *ρ*_*e*_ and has been explored by many groups [27–29]. Generally, DECT methods decompose the data of two CT scans of different energies into *ρ*_*e*_ and effective atomic number (EAN). In the case of proton therapy, EAN is then related to mean ionization potential/average ionization energy (*I*_*m*_) or I-value. From a physics perspective, this decomposition elegantly solves the problem of no one-to-one relationship between *ρ*_*e*_ and HU but there may still be a bit of ambiguity in the determination of mean ionization potential/average ionization energy (*I*_*m*_) with many proposed solutions to relate EAN to *I*_*m*_ [28, 30, 31]. Molecularly based treatments, such as in the present work, have even been proposed as a methodology to accurately calculate *I*_*m*_ [32]. Yet the promise of DECT has been overshadowed by issues related to DECT’s increased sensitivity to noise [33, 34] and beam hardening effects [35] with the end result being more modest clinical improvements in determination of *ρ*_*e*_ and stopping power for proton therapy applications. Accurate determination of *ρ*_*e*_ is highly important in proton therapy as *ρ*_*e*_ is linear with stopping power and thus any uncertainties in *ρ*_*e*_ are directly proportional to uncertainties in beam position (along the beam axis). The methods proposed in this work may help add another tool in the growing toolkit of methods to more accurately convert HU to *ρ* and *ρ*_*e*_ for more accurate radiation therapy dose calculation.

## Conclusions

In this study, it is shown that kVCT HU is not one-to-one in its relationship with *ρ* and *ρ*_*e*_. It was found that this ambiguous relationship can be resolved with more information as there is a multi-dimensional relationship between molecular (water, lipid, protein, and mineral) composition, HU, *ρ*, and *ρ*_*e*_. A novel model-based relationship between molecular (water, lipid, protein, and mineral) composition, HU, *ρ*, and *ρ*_*e*_ was developed and shown to be accurate provided that molecular composition is known. Further development work in modeling the molecular composition of tissues may be required to fully realize the improved accuracy of this methodology.

## References

[1] Schlegel W, Bortfeld T, Grosu A. New technologies in radiation oncology. Berlin; London: Springer; 2006 464 p. p.

[2] Ainsley CG, Yeager CM. Practical considerations in the calibration of CT scanners for proton therapy. J Appl Clin Med Phys. 2014;15(3):4721 Epub 2014/06/04. 10.1120/jacmp.v15i3.4721 24892347 PMC5711046

[3] Yang M, Virshup G, Mohan R, Shaw CC, Zhu XR, Dong L. Improving accuracy of electron density measurement in the presence of metallic implants using orthovoltage computed tomography. Med Phys. 2008;35(5):1932–41. Epub 2008/06/20. 10.1118/1.2905030 . 18561669

[4] Fang R, Mazur T, Mutic S, Khan R. The impact of mass density variations on an electron Monte Carlo algorithm for radiotherapy dose calculations. Physics and Imaging in Radiation Oncology. 2018;8:1–7. 10.1016/j.phro.2018.10.002 PMC7807677 33458409

[5] Langen KM, Meeks SL, Poole DO, Wagner TH, Willoughby TR, Kupelian PA, et al The use of megavoltage CT (MVCT) images for dose recomputations. Phys Med Biol. 2005;50(18):4259–76. Epub 2005/09/09. 10.1088/0031-9155/50/18/002 . 16148392

[6] Ruchala KJ, Olivera GH, Schloesser EA, Hinderer R, Mackie TR. Calibration of a tomotherapeutic MVCT system. Phys Med Biol. 2000;45(4):N27–36. Epub 2000/05/05. 10.1088/0031-9155/45/4/404 . 10795996

[7] Parker RP, Hobday PA, Cassell KJ. The direct use of CT numbers in radiotherapy dosage calculations for inhomogeneous media. Phys Med Biol. 1979;24(4):802–9. Epub 1979/07/01. 10.1088/0031-9155/24/4/011 . 472014

[8] Henson PW, Fox RA. The electron density of bone for inhomogeneity correction in radiotherapy planning using CT numbers. Phys Med Biol. 1984;29(4):351–9. Epub 1984/04/01. 10.1088/0031-9155/29/4/005 . 6718488

[9] Dobbs HJ, Parker RP, Hodson NJ, Hobday P, Husband JE. The use of CT in radiotherapy treatment planning. Radiother Oncol. 1983;1(2):133–41. Epub 1983/11/01. 10.1016/s0167-8140(83)80016-4 . 6680218

[10] Stutzel J, Oelfke U, Nill S. A quantitative image quality comparison of four different image guided radiotherapy devices. Radiother Oncol. 2008;86(1):20–4. Epub 2007/11/23. 10.1016/j.radonc.2007.10.035 . 18031854

[11] Meeks SL, Harmon JF Jr., Langen KM, Willoughby TR, Wagner TH, Kupelian PA. Performance characterization of megavoltage computed tomography imaging on a helical tomotherapy unit. Med Phys. 2005;32(8):2673–81. Epub 2005/10/01. 10.1118/1.1990289 . 16193798

[12] Heymsfield S. Human body composition. 2nd ed Champaign, IL: Human Kinetics; 2005 xii, 523 p. p.

[13] Wang ZM, Pierson RN Jr., Heymsfield SB. The five-level model: a new approach to organizing body-composition research. Am J Clin Nutr. 1992;56(1):19–28. Epub 1992/07/11. 10.1093/ajcn/56.1.19 . 1609756

[14] Berger MJ, Hubbell JH, Seltzer SM, Chang J, Coursey JS, Sukumar R, et al XCOM: Photon Cross Section Database (version 1.5) Gaithersburg, MD: National Institute of Standards and Technology; 2010 [2020, March 26]. Available from: http://physics.nist.gov/xcom.

[15] Punnoose J, Xu J, Sisniega A, Zbijewski W, Siewerdsen JH. Technical Note: spektr 3.0-A computational tool for x-ray spectrum modeling and analysis. Med Phys. 2016;43(8):4711 Epub 2016/08/05. 10.1118/1.4955438 27487888 PMC4958109

[16] Siewerdsen JH, Waese AM, Moseley DJ, Richard S, Jaffray DA. Spektr: a computational tool for x-ray spectral analysis and imaging system optimization. Med Phys. 2004;31(11):3057–67. Epub 2004/12/14. 10.1118/1.1758350 . 15587659

[17] Jeraj R, Mackie TR, Balog J, Olivera G, Pearson D, Kapatoes J, et al Radiation characteristics of helical tomotherapy. Med Phys. 2004;31(2):396–404. Epub 2004/03/06. 10.1118/1.1639148 . 15000626

[18] Zhou H, Keall PJ, Graves EE. A bone composition model for Monte Carlo x-ray transport simulations. Med Phys. 2009;36(3):1008–18. Epub 2009/04/22. 10.1118/1.3077129 . 19378761

[19] White DR, Woodard HQ, Hammond SM. Average soft-tissue and bone models for use in radiation dosimetry. Br J Radiol. 1987;60(717):907–13. Epub 1987/09/01. 10.1259/0007-1285-60-717-907 . 3664185

[20] Woodard HQ, White DR. Bone models for use in radiotherapy dosimetry. Br J Radiol. 1982;55(652):277–82. Epub 1982/04/01. 10.1259/0007-1285-55-652-277 . 7066638

[21] ICRU. Tissue substitutes in radiation dosimetry and measurement. Bethesda, Md., U.S.A.: International Commission on Radiation Units and Measurements; 1989 vii, 189 p. p.

[22] White DR, Widdowson EM, Woodard HQ, Dickerson JW. The composition of body tissues (II). Fetus to young adult. Br J Radiol. 1991;64(758):149–59. Epub 1991/02/01. 10.1259/0007-1285-64-758-149 . 2004206

[23] Woodard HQ, White DR. The composition of body tissues. Br J Radiol. 1986;59(708):1209–18. Epub 1986/12/01. 10.1259/0007-1285-59-708-1209 . 3801800

[24] Scholey JE, Chandramohan D, Naren T, Liu W, Larson PEZ, Sudhyadhom A. Technical note: A methodology for improved accuracy in stopping power estimation using MRI and CT. Med Phys. 2020 Epub 2020/10/28. 10.1002/mp.14555 . 33107997 PMC8717393

[25] Aamir R, Chernoglazov A, Bateman CJ, Butler APH, Butler PH, Anderson NG, et al MARS spectral molecular imaging of lamb tissue: data collection and image analysis. Journal of Instrumentation. 2014;9(02):P02005–P. 10.1088/1748-0221/9/02/p02005

[26] Balagopal A, Kazemifar S, Nguyen D, Lin MH, Hannan R, Owrangi A, et al Fully automated organ segmentation in male pelvic CT images. Phys Med Biol. 2018;63(24):245015 Epub 2018/12/14. 10.1088/1361-6560/aaf11c . 30523973

[27] Landry G, Reniers B, Granton PV, van Rooijen B, Beaulieu L, Wildberger JE, et al Extracting atomic numbers and electron densities from a dual source dual energy CT scanner: experiments and a simulation model. Radiother Oncol. 2011;100(3):375–9. Epub 2011/09/20. 10.1016/j.radonc.2011.08.029 . 21924780

[28] Hunemohr N, Paganetti H, Greilich S, Jakel O, Seco J. Tissue decomposition from dual energy CT data for MC based dose calculation in particle therapy. Med Phys. 2014;41(6):061714 Epub 2014/06/01. 10.1118/1.4875976 24877809 PMC4032427

[29] Tsukihara M, Noto Y, Hayakawa T, Saito M. Conversion of the energy-subtracted CT number to electron density based on a single linear relationship: an experimental verification using a clinical dual-source CT scanner. Phys Med Biol. 2013;58(9):N135–44. Epub 2013/04/11. 10.1088/0031-9155/58/9/N135 . 23571116

[30] Yang M, Virshup G, Clayton J, Zhu XR, Mohan R, Dong L. Theoretical variance analysis of single- and dual-energy computed tomography methods for calculating proton stopping power ratios of biological tissues. Phys Med Biol. 2010;55(5):1343–62. Epub 2010/02/11. 10.1088/0031-9155/55/5/006 . 20145291

[31] Bourque AE, Carrier JF, Bouchard H. A stoichiometric calibration method for dual energy computed tomography. Phys Med Biol. 2014;59(8):2059–88. Epub 2014/04/04. 10.1088/0031-9155/59/8/2059 . 24694786

[32] Sudhyadhom A. Determination of mean ionization potential using magnetic resonance imaging for the reduction of proton beam range uncertainties: theory and application. Phys Med Biol. 2017;62(22):8521–35. Epub 2017/10/28. 10.1088/1361-6560/aa8d9e . 29077570

[33] Bar E, Lalonde A, Royle G, Lu HM, Bouchard H. The potential of dual-energy CT to reduce proton beam range uncertainties. Med Phys. 2017;44(6):2332–44. Epub 2017/03/16. 10.1002/mp.12215 . 28295434

[34] Lee HHC, Li B, Duan X, Zhou L, Jia X, Yang M. Systematic analysis of the impact of imaging noise on dual-energy CT-based proton stopping power ratio estimation. Med Phys. 2019;46(5):2251–63. Epub 2019/03/19. 10.1002/mp.13493 30883827 PMC6510613

[35] Li B, Lee HC, Duan X, Shen C, Zhou L, Jia X, et al Comprehensive analysis of proton range uncertainties related to stopping-power-ratio estimation using dual-energy CT imaging. Phys Med Biol. 2017;62(17):7056–74. Epub 2017/07/06. 10.1088/1361-6560/aa7dc9 28678019 PMC5736379

